# Association of *COMT* and *COMT*-*DRD2* interaction with creative potential

**DOI:** 10.3389/fnhum.2014.00216

**Published:** 2014-04-14

**Authors:** Shun Zhang, Muzi Zhang, Jinghuan Zhang

**Affiliations:** Department of Psychology, Shandong Normal UniversityJinan, China

**Keywords:** creativity, creative potential, divergent thinking, dopamine, *COMT*, *DRD2*, gene–gene interaction

## Abstract

Several lines of evidence suggest that genes involved in dopamine (DA) transmission may contribute to creativity. Among these genes, the catechol-O-methyltransferase gene (*COMT*) and the dopamine D2 receptor gene (*DRD2*) are the most promising candidates. Our previous study has revealed evidence for the involvement of *DRD2* in creative potential. The present study extended our previous study by systematically exploring the association of *COMT* with creative potential as well as the interaction between *COMT* and *DRD*2. Twelve single nucleotide polymorphisms (SNPs) covering *COMT* were genotyped in 543 healthy Chinese college students whose creative potentials were assessed by divergent thinking tests. Single SNP analysis showed that rs174697 was nominally associated with verbal originality, two SNPs (rs737865 and rs5993883) were nominally associated with figural fluency, and two SNPs (rs737865 and rs4680) were nominally associated with figural originality. Haplotype analysis showed that, the TCT and CCT haplotype (rs737865-rs174675-rs5993882) were nominally associated with figural originality, and the TATGCAG and CGCGGGA haplotype (rs4646312-rs6269-rs4633-rs6267-rs4818-rs4680-rs769224) were nominally associated with figural originality and verbal flexibility, respectively. However, none of these nominal findings survived correction for multiple testing. Gene–gene interaction analysis identified one significant four-way interaction of rs174675 (*COMT*), rs174697 (*COMT*), rs1076560 (*DRD2*), and rs4436578 (*DRD2*) on verbal fluency, one significant four-way interaction of rs174675 (*COMT*), rs4818 (*COMT*), rs1076560 (*DRD2*), and rs4648317 (*DRD2*) on verbal flexibility, and one significant three-way interaction of rs5993883 (*COMT*), rs4648319 (*DRD2*), and rs4648317 (*DRD2*) on figural flexibility. In conclusion, the present study provides nominal evidence for the involvement of *COMT* in creative potential and suggests that DA related genes may act in coordination to contribute to creativity.

## Introduction

Creativity refers to the ability to produce something that is both novel and useful (Sternberg and Lubart, 1999). It is closely related to human development and achievement at both the individual and societal level. Despite its importance, the underlying mechanism of how creativity works is not completely understood. The need for a deeper understanding of the biological correlates of creative cognition has inspired a great number of neuroscience and cognitive studies. Findings from these studies generally support a critical involvement of dopamine (DA) transmission in the cognitive process of creativity (Flaherty, 2005; Chermahini and Hommel, 2010; Takeuchi et al., 2010). Hence, genes involved in DA transmission have been of particular interest to explain individual differences in creativity. Amongst these genes, the catechol-O-methyltransferase gene (*COMT*) and the dopamine D2 receptor gene (*DRD2*) have been studied most extensively.

The *COMT* gene is located on chromosome 22q11. The enzyme encoded by this gene is involved in the inactivation of the catecholamine neurotransmitters (DA, adrenalin, and noradrenalin) (Axelrod, 1957) and is the main factor controlling DA levels in the prefrontal cortex (PFC). The *DRD2* gene, located on chromosome 11q22-23, encodes one of five DA receptors and plays an important role in mediating synaptic DA signaling. Variants of these two genes have been repeatedly implicated in creativity related cognitive functions, such as working memory and cognitive control (Egan et al., 2001; Bruder et al., 2005; Zhang et al., 2007; Diaz-Asper et al., 2008; Bertolino et al., 2010; Colzato et al., 2010, 2013).

By employing divergent thinking (DT) tests as a measure of creative potential, several attempts have been made to identify *COMT* and *DRD2* related genetic variants associated with creativity. Reuter et al. (2006) investigated the influence of *COMT* VAL158MET polymorphism (rs4680) and *DRD2/ANKK1* Taq IA polymorphism (rs1800497) on creative potential, and demonstrated that *DRD2/ANKK1* rs1800497 was associated with total creativity score. Runco et al. (2011) further extended Reuter et al.'s work by investigating the effects of *COMT* rs4680 and *DRD2/ANKK1* rs1800497 on the three common indexes (fluency, originality, and flexibility) of both verbal and figural DT tests. However, the result indicated that only *COMT* rs4680 was associated with fluency, and neither of these two genetic variants was related to originality or flexibility when controlling for the significant effect of fluency. Although these studies provide important insight into the underlying genetic basis of creativity, it is important to note that, for both *COMT* and *DRD2*, only one genetic variant from each gene was investigated. Thus, it is not clear whether there are other genetic variants of these two genes associated with creative potential.

To fill this gap in the literature, our recent study comprehensively explored the associations of *DRD2* related genetic polymorphisms with creative potential in the Han Chinese population and found several previously unrevealed *DRD2* SNPs and haplotypes associated with DT fluency, originality and flexibility (Zhang et al., 2014). This suggests that a more detailed examination of the genetic variants covering these genes will provide additional valuable information about the effects of these genes on creative potential. Therefore, by using the same approach in the same sample, the present study aimed to systematically investigate the associations of *COMT* related genetic polymorphisms with creative potential.

Furthermore, there is evidence suggesting a nonlinear relationship between DA and creative potential (Chermahini and Hommel, 2010, 2012). This indicates that interactions among DA related genes may contribute to creativity potential. By reanalyzing Runco et al.'s data, Murphy et al. (2013) recently investigated the interaction between *COMT* and *DRD2*. However, because no genetic variant was further genotyped, Murphy et al.'s analysis was confined to the interaction between *COMT* rs4680 and *DRD2/ANKK1* rs1800497, the interaction between *COMT* and *DRD2* on creative potential remains largely unknown and needs to be further assessed. Thus, the present study further extended the literature as well as our previous study by systematically exploring the interaction between *COMT* and *DRD2*.

## Methods

### Participants and procedure

The main characteristics of participants and study procedure have been described previously (Zhang et al., 2014). In summary, the sample consisted of 543 unrelated healthy Chinese college students (185 males and 358 females, with a mean age of 18.94 years, *SD* = 0.84) from Shandong Normal University. All participants were of Han Chinese descendants and with no self-reported history of neurological and psychiatric disorder. This study was approved by the Institutional Review Board of Shandong Normal University, and all study participants gave written informed consent. Participants first completed the psychometric tests, and then peripheral venous blood samples were collected for genotyping.

### SNP selection

In order to ensure a full genetic coverage of *COMT*, nine tag SNPs (rs737865, rs174675, rs5993882, rs5993883, rs4646312, rs6267, rs4680, rs769224, and rs174697) were selected from HapMap (http://hapmap.ncbi.nlm.nih.gov) genotype data for the CHB population (Data Rel 27 Phase II + III, Feb09, on NCBI B36 assembly, dbSNP b126) by applying the Tagger program implemented in Haploview v4.2 software (Barrett et al., 2005) with parameters of minor allele frequency (MAF) > 5% and pair-wise *r*^2^ > 0.8. The nine tag SNPs captured 80% of common alleles (MAF > 5%) within the genomic region of *COMT* (chr22:18309309..18336528, based on NCBI Genome Build 36.3) with a mean maximal *r*^2^ of 0.966. In addition to the nine tag SNPs, three putative functional SNPs (rs6269, rs4633, and rs4818), all of which were at coding regions, were also genotyped. Table 1 summarizes the final set of genotyped *COMT* SNPs. The selection of *DRD2* SNPs has been described in detail previously (Zhang et al., 2014).

**Table 1:** **Descriptive statistics and inter-correlations^a^**.

**Measure**	**Cronbach's α**	**M (*SD*)**			**1**	**2**	**3**	**4**	**5**	**6**
		**Total**	**Male**	**Female**						
1. Verbal fluency	0.85	8.25 (2.86)	7.43 (2.80)	8.67 (2.80)^**^		0.84^**^	0.78^**^	0.73^**^	0.69^**^	0.60^**^
2. Verbal originality	0.75	2.48 (1.68)	2.27 (1.71)	2.59 (1.65)^*^			0.65^**^	0.62^**^	0.63^**^	0.51^**^
3. Verbal flexibility	0.70	4.40 (1.08)	4.25 (1.21)	4.48 (1.00)^*^				0.59^**^	0.55^**^	0.55^**^
4. Figural fluency	0.88	10.06 (4.25)	8.40 (3.99)	10.91 (4.12)^**^					0.93^**^	0.82^**^
5. Figural originality	0.83	4.90 (3.05)	4.05 (2.79)	5.34 (3.09)^**^						0.75^**^
6. Figural flexibility	0.69	5.10 (1.27)	4.69 (1.39)	5.31 (1.15)^**^						

a Statistical significance was determined by permutation testing.

* P < 0.05

** P < 0.01.

### Genotyping

Methods for DNA extraction and genotyping have been described previously (Zhang et al., 2014). Briefly, genomic DNA was extracted from peripheral venous blood sample using the QIAamp DNA Mini Kit (Qiagen, Valencia, CA, USA). Genotypings for all SNPs were performed at Beijing Genomics Institute-Shenzhen (BGI-Shenzhen, CityShenzhen, China) by using the Sequenom® MassARRAY® iPLEX system (Sequenom, San Diego, StateCA, USA). For quality control, 5% random DNA samples were genotyped twice for each SNP to calculate genotyping error. The genotyping accuracy was 100%.

### Creative potential measures—DT tests

As previously described (Zhang et al., 2014), verbal and figural DT tests (each containing three tasks) selected from Runco Creativity Assessment Battery (rCAB; Creativity Testing Service, Bishop, GA, USA) were used to assess creative potential. The rCAB is comparable to other assessments of fluency, originality, and flexibility (e.g., Wallach and Kogan, 1965). In verbal DT test, participants were asked to list as many different uses as they could for three common subjects (toothbrush, tire, and spoon). In figural DT test, three line drawings were represented and participants were asked to list as many things as each line drawing could be. Four minutes was allowed for each task. All DT tasks were scored for fluency, flexibility, and originality according to the guideline of Creativity Testing Service. Briefly, fluency score was obtained by counting the number of unduplicated responses given by each participant. Originality score was calculated by counting the number of unusual responses (responses given by less than 5% of the sample). In order to score flexibility, a category list was first generated for each task, and the flexibility score was the number of different categories used in one participant's responses. For each task, two trained raters (both were psychology graduate students from Shandong Normal University) were engaged to score all the responses. The inter-rater reliabilities for all the six DT scores were higher than 0.95.

### Statistical analysis

To adjust for confounding factors and the effect of fluency (Hocevar, 1980; Runco and Albert, 1985), covariate-adjusted standardized residuals of DT scores were first calculated using multivariate linear regression. Specifically, for verbal and figural fluency, the covariate adjusted was gender. For verbal originality and flexibility, the covariates included gender and verbal fluency score. For figural originality and flexibility, the covariates included gender and figural fluency score. These residuals were used in association analysis described below.

Hardy–Weinberg equilibrium was tested by Fisher's exact test using Plink v1.07 software (Purcell et al., 2007). Single SNP analysis under three different genetic models (dominant, additive and recessive) was performed using linear regression in Plink. For SNP with minor allele homozygotes <5%, only the dominant model was tested. Pair-wise linkage disequilibrium (LD) and haplotype blocks were assessed by Haploview. Association analysis for the identified haplotype blocks was performed using linear regression in Plink. Haplotypes with estimated frequency <5% were excluded from the analysis. For both single SNP and haplotype analysis, empirical *P*-values were computed by using the maxT permutation procedure implemented in Plink with 10,000 permutations.

Gene–gene interactions among genetic variants of *COMT* and *DRD2* were analyzed using the Quantitative Multifactor Dimensionality Reduction (QMDR) approach implemented in MDR v3.0.2 software (Hahn et al., 2003). The QMDR approach is an extension of the original MDR approach to handle quantitative traits, and has been described in detail previously (Gui et al., 2013). In brief, the QMDR approach shared the same reduction strategy and cross-validation procedure as the MDR approach and the difference is that, the QMDR approach handles quantitative data by modifying MDR's constructive induction algorithm to use a *t*-test. In QMDR, by comparing the mean outcome of each multi-locus genotype combination to the overall mean outcome, all possible multi-locus genotype combinations were first combined into two different groups (high-level or low-level). Then the mean outcome of the high-level and the low-level group was compared using a *t*-test and the *t*-test statistics from the training set and testing set were defined as the scores to determine the best interaction model. In the present study, to reduce redundancy, SNPs in strong LD (*r*^2^>0.8) with another essayed SNP were first excluded, and then an exhaustive search of all possible two-, three- and four-way interactions among the remaining 18 SNPs (nine from *COMT* and nine from *DRD2*) was performed using QMDR. The best overall model was determined by 10-fold cross-validation and the empirical *P*-values were computed by 1000 permutations.

## Results

Table 2 shows the descriptive statistics and inter-correlations matrix. All six DT scores demonstrated reliability and were highly inter-correlated. Female participants performed better in DT tests than male participants.

**Table 2:** **Characteristics of the genotyped *COMT* SNPs**.

**SNP^a^**	**Position^b^**	**Location**	**Allele (minor/major)**	**MAF (%)**
rs737865	18310121	Intron 1	C/T	29.8
rs174675	18314051	Intron 1	T/C	39.0
rs5993882	18317533	Intron 1	G/T	12.2
rs5993883	18317638	Intron 1	G/T	41.5
rs4646312	18328337	Intron 1	C/T	37.0
rs6269	18329952	Exon 3	G/A	37.4
rs4633	18330235	Exon 3	T/C	26.2
rs6267	18330263	Exon 3	T/G	7.5
rs4818	18331207	Exon 4	G/C	36.7
rs4680	18331271	Exon 4	A/G	26.7
rs769224	18331804	Exon 5	A/G	6.6
rs174697	18333832	Intron 5	A/G	33.4

MAF, minor allele frequency.

a SNPs are listed down the column in sequential order from the 5′ end to the 3′ end of the sense strand of COMT.

b Physical position is based on NCBI Genome Build 36.3.

### Single SNP and haplotype association

**Figure 1:**
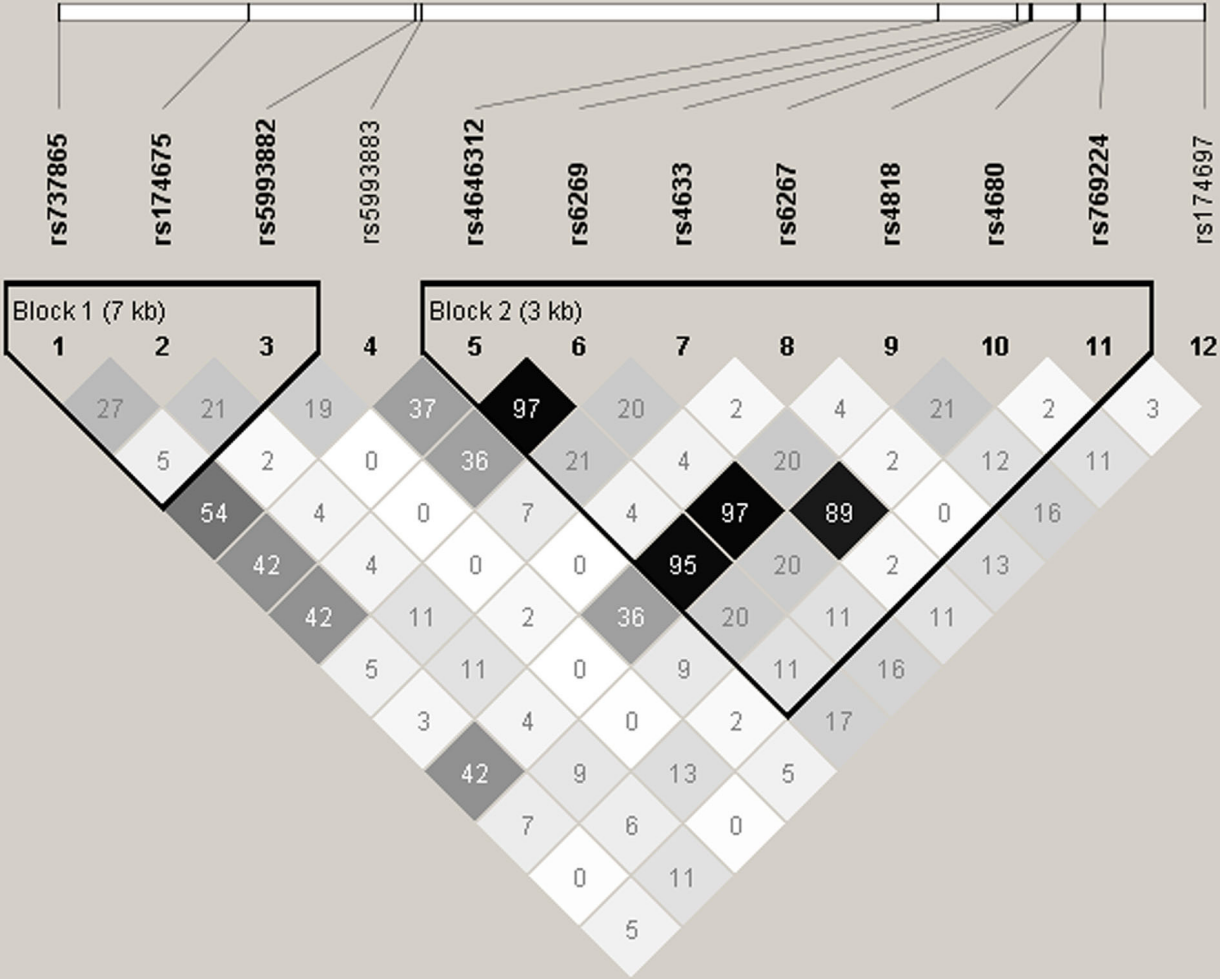
**Linkage disequilibrium (LD) pattern of the 12 *COMT* SNPs analyzed in the present study**. Numbers in squares designate the degree of LD (*r*^2^) between any two SNPs. LD blocks were defined using the solid spine of LD algorithm implemented in Haploview.

No significant deviations from Hardy–Weinberg equilibrium were observed for the 12 *COMT* SNPs (data not shown). Table 3 summarizes the results of single SNP analysis. In particular, for verbal DT test, rs174697 showed nominal association with verbal originality under the additive model. For figural DT test, two SNPs (rs737865 and rs5993883) were nominally associated with figural fluency under the dominant model, and two SNPs (rs737865 and rs4680) showed nominal associations with figural originality under the additive model. The LD patterns of the 12 *COMT* SNPs are shown in Figure 1. Two haplotype blocks were defined using the solid spine of LD algorithm, and were further tested for association with creative potential. The 3-SNP haplotype block (Block 1) displayed nominal association with figural originality. Specifically, the TCT and CCT haplotype (rs737865-rs174675-rs5993882) were nominally associated with figural originality. For the 7-SNP haplotype block, although the global test did not reveal any association, the TATGCAG and CGCGGGA haplotype (rs4646312-rs6269-rs4633-rs6267-rs4818-rs4680-rs769224) were found to be nominally associated with figural originality and verbal flexibility, respectively, (Table 4). None of these nominal single SNP and haplotype associations withstood correction for multiple testing (data not shown).

**Table 3:** **Selected results of single SNP analysis for creative potential^a^**.

**SNP**	**Verbal originality^b^**				**Figural fluency^c^**				**Figural originality^d^**			
	**Model**	***B***	***t***	***P***_***emp***_	**Model**	***B***	***t***	***P***_***emp***_	**Model**	***B***	***t***	***P _emp_***
rs737865	Dominant	−0.079	−0.918	0.354	Dominant	0.189	2.21	**0.028**	Additive	−0.163	−2.49	**0.015**
rs174675	Dominant	−0.093	−1.04	0.299	Recessive	−0.076	−0.621	0.529	Recessive	0.096	0.782	0.435
rs5993882	Dominant	−0.037	−0.361	0.721	Dominant	0.013	0.124	0.898	Dominant	0.091	0.892	0.374
rs5993883	Dominant	−0.067	−0.743	0.460	Dominant	0.190	2.11	**0.035**	Dominant	−0.153	−1.70	0.092
rs4646312	Additive	0.071	1.17	0.245	Additive	0.094	1.54	0.117	Dominant	−0.143	−1.64	0.103
rs6269	Additive	0.072	1.19	0.234	Additive	0.096	1.57	0.105	Dominant	−0.141	−1.62	0.107
rs4633	Dominant	0.034	0.391	0.697	Recessive	−0.264	−1.74	0.079	Dominant	0.159	1.85	0.065
rs6267	Dominant	−0.117	−0.951	0.339	Dominant	−0.068	−0.550	0.578	Dominant	−0.002	−0.01	0.986
rs4818	Additive	0.064	1.05	0.296	Additive	0.104	1.70	0.081	Dominant	−0.159	−1.83	0.066
rs4680	Recessive	−0.022	−0.149	0.882	Recessive	−0.199	−1.33	0.179	Additive	0.144	2.20	**0.030**
rs769224	Dominant	0.168	1.32	0.187	Dominant	0.027	0.214	0.835	Dominant	0.019	0.152	0.881
rs174697	Additive	−0.124	−2.02	**0.043**	Dominant	0.020	0.236	0.811	Dominant	−0.149	−1.74	0.082

a Covariates-adjusted DT scores were analyzed for association by linear regression under three (additive, dominant, and recessive) genetic models, and empirical P-values (P_emp_) were obtained by 10,000 permutations. For rs5993882, rs6267, and rs769224 (minor allele homozygotes <5%), only the dominant genetic model was tested. Only the best result and the corresponding genetic model are shown for each SNP. The significance values <0.05 are indicated in bold.

b Adjusted for gender and verbal fluency.

c Adjusted for gender.

d Adjusted for gender and figural fluency.

**Table 4:** **Selected results of haplotype analysis for creative potential^a^**.

**Block**	**Haplotype**	**Frequencies (%)**	**Verbal flexibility^d^**			**Figural originality^e^**		
			***B***	***t***	***P_emp_***	***B***	***t***	***P******_emp_***
Block 1^b^	TCT	31.3	−0.031	0.242	0.626	0.128	4.11	**0.048**
	CCT	29.7	0.064	0.956	0.323	−0.163	6.23	**0.014**
	TTT	26.8	−0.005	0.006	0.939	−0.029	0.175	0.679
	TTG	12.2	−0.053	0.316	0.577	0.105	1.27	0.265
	Global test				0.779			**0.045**
Block 2^c^	CGCGGGG	29.7	0.031	0.211	0.647	−0.122	3.31	0.068
	TACGCGG	28	0.030	0.212	0.642	−0.029	0.200	0.650
	TATGCAG	25.3	0.085	1.64	0.204	0.140	4.41	**0.039**
	TACTCGG	7.5	−0.092	0.655	0.419	0.019	0.029	0.868
	CGCGGGA	6.6	−0.249	4.03	**0.042**	0.029	0.054	0.820
	Global test				0.232			0.224

a Haplotype frequencies were estimated using the expectation-maximization (EM) algorithm in Plink and linear regression was used to estimate haplotype-specific effect on covariates-adjusted DT scores. An omnibus test was also employed to obtain a global P-value for the haplotype block. Empirical P-values (P_emp_) were obtained by 10,000 permutations. Rare haplotypes (estimated frequency <5%) were excluded from the analysis. The significance values <0.05 are indicated in bold.

b The order of SNPs in block 1 was rs737865, rs174675, and rs5993882.

c The order of SNPs in block 2 was rs4646312, rs6269, rs4633, rs6267, rs4818, rs4680, and rs769224.

d Adjusted for gender and verbal fluency.

e Adjusted for gender and figural fluency.

### Gene–gene interaction

Table 5 summarizes the results of QMDR analysis for DT verbal fluency, verbal flexibility, and figural flexibility. For verbal fluency, the overall best model was a four-way interaction of rs174675 (*COMT*), rs174697 (*COMT*), rs1076560 (*DRD2*), and rs4436578 (*DRD2*). These four SNPs had a training score of 8.60 and a testing score of 5.62. The cross-validation consistency of this model was 10/10. The 1000 permutation testing revealed a significant empirical *P*-value of 0.002. For verbal flexibility, the overall best model was a four-way interaction of rs174675 (*COMT*), rs4818 (*COMT*), rs1076560 (*DRD2*) and rs4648317 (*DRD2*), with a training score of 7.97, a testing score of 4.76, and a cross-validation consistency of 10/10. Empirical *P*-value of this model was of 0.005. For figural flexibility, the overall best model was a three-way interaction of rs5993883 (*COMT*), rs4648319 (*DRD2*), and rs4648317 (*DRD2*). This model showed a training score of 5.65, a testing score of 3.97, a cross-validation consistency of 10/10, and an empirical *P*-value of 0.027. No statistically significant interactions were reported for other DT scores.

**Table 5:** **Selected results of gene–gene interaction analysis for creative potential^a^**.

**DT score**	**Combination**	**CVC**	**Training score**	**Testing score**	***P***_***emp***_
Verbal fluency^b^	rs4436578 (*COMT*), rs6279 (*DRD2*)	3/10	4.03	−0.176	0.817
	rs174675 (*COMT*), rs174697 (*COMT*), rs1076560 (*DRD2*)	3/10	5.53	−0.971	0.943
	rs174675 (*COMT*), rs174697 (*COMT*), rs1076560 (*DRD2*), rs4436578 (*DRD2*)	10/10	8.60	5.62	**0.002**
Verbal flexibility^c^	rs4818 (*COMT*), rs1076560 (*DRD2*)	4/10	3.83	−0.427	0.871
	rs4818 (*COMT*), rs1076560 (*DRD2*), rs4648317 (*DRD2*)	3/10	5.58	0.368	0.688
	rs174675 (*COMT*), rs4818 (*COMT*), rs1076560 (*DRD2*), rs4648317 (DR*D2*)	10/10	7.97	4.76	**0.005**
Figural flexibility^d^	rs174675 (*COMT*), rs6279(*DRD*2)	4/10	3.45	−0.637	0.906
	rs5993883 (*COMT*), rs4648319 (*DRD2*), rs4648317 (*DRD2*)	10/10	5.65	3.97	**0.027**
	rs5993883 (*COMT*), rs174697 (*COMT*), rs4648319 (*DRD2*), rs4648317 (*DRD2*)	7/10	7.67	0.987	0.500

CVC, cross-validation consistency.

a Gene–gene interaction analysis for covariates-adjusted DT scores was performed by QMDR. An exhaustive search of all possible two-, three- and four-way interactions was performed among the 18 COMT and DRD2 SNPs. For COMT, the SNPs included were rs737865, rs174675, rs5993882, rs5993883, rs6267, rs4818, rs4680, rs769224, and rs174697. For DRD2, the SNPs included were rs6279, rs6277, rs1076560, rs4436578, rs4648319, rs4245148, rs4648317, rs1799732, and rs1799978. Empirical P-values (P_emp_) were obtained by 1000 permutations. The significance values <0.05 are indicated in bold.

b Adjusted for gender.

c Adjusted for gender and verbal fluency.

d Adjusted for gender and figural fluency.

## Discussion

Given the hypothesized role of DA in the neurobiological underpinnings of creativity as well as the crucial role of COMT in the regulation of DA transmission, *COMT* is among the many highly plausible functional candidate genes for creativity.

To date there have been only two published studies that have examined the association of *COMT* with creative potential. Both of these studies focused on rs4680. The rs4680 is among one of the most frequently studied *COMT* polymorphisms. The G to A substitution at this position translates into a substitution of methionine (MET) for valine (VAL) at codon 158, which results in 3- to 4-fold difference in COMT enzymatic activity (Lotta et al., 1995; Lachman et al., 1996; Chen et al., 2004). The A allele has lower enzymatic activity than the G allele, thereby leading to less efficient degradation of DA and higher DA level in the synaptic cleft (Chen et al., 2004). In a sample of 92 Caucasians, Reuter et al. (2006) first investigated the association of rs4680 with creative potential, and reported no association of rs4680 with creative potential. However, because Reuter et al. employed a composite index as a measure of creative potential, the particular contribution of rs4680 to the three core dimensions of DT (fluency, originality, and flexibility) could not be determined. Runco et al. (2011) extended this work by examining the specific effect of rs4680 on both verbal and figural DT fluency, originality and flexibility, and found rs4680 was associated with DT fluency. However, it should be noted that, both studies used small sample sizes, and neither of them examined other genetic variants of *COMT*.

The present study extended previous research by conducting a detailed association analysis of *COMT* with creative potential in a relatively large Chinese sample using 12 SNPs spanning the gene. Single SNP analysis identified four SNPs nominally associated with creative potential. Among the four SNPs, the previously reported rs4680 was found to be nominally associated with figural originality, with the A allele associated with higher figural originality score. This result is in line with previous findings that the A allele of rs4680 is associated with better creativity related cognitive performance (such as working memory) (Egan et al., 2001; Malhotra et al., 2002; Bruder et al., 2005; Barnett et al., 2007; Aguilera et al., 2008; Diaz-Asper et al., 2008) and creativity related personality traits (such as novelty seeking) (Tsai et al., 2004; Golimbet et al., 2007; Davila et al., 2013). However, it is also important to note that, the previously reported association of rs4680 with DT fluency was not replicated in the present study. One possible explanation for this discrepancy could be the genetic heterogeneity between Han Chinese and Caucasians. Besides rs4680, three intronic SNPs also showed nominal associations with creative potential. The rs737865 was nominally associated with both figural fluency and figural originality, while rs5993883 and rs174697 were nominally associated with figural fluency and verbal originality, respectively. For these intronic SNPs, because few studies have been conducted to examine their functions, there exists less supporting evidence for the observed associations. However, previous research has shown that intronic variants may play critical roles in the regulation of gene expression (Nackley et al., 2006; Wang and Cooper, 2007; Zhang et al., 2007). It is possible that these intronic SNPs might play a role in regulating *COMT* expression. Further functional analysis is needed to test this hypothesis. Another possibility is that these SNPs might be in high LD with other unidentified causative variants. In addition to single SNP analysis, haplotype analysis also identified three haplotypes (the TCT and CCT haplotype of Block1, and the TATGCAG haplotype of Block 2) nominally associated with figural originality, as well as one haplotype (the CGCGGGA haplotype of Block 2) nominally associated with verbal flexibility. However, caution should be exercised when interpreting these results, since these nominally significant single SNP and haplotype associations would not survive correction for multiple testing. Therefore, these results should only be considered as suggestive, replication by independent studies is necessary to confirm these nominal findings.

The nonlinear relationship between DA and creative potential suggests that genes involved in DA transmission may interact to affect creative potential (Chermahini and Hommel, 2010, 2012). Our previous study found some suggestive evidence for the association of *DRD2* with creative potential. Therefore, the present study further extended our previous study by systematically exploring the interaction between *COMT* and *DRD2*. For these two genes, a total of 18 SNPs were included and examined for all their possible two-, three- and four-way interactions using the QMDR approach. Linear regression or MANOVA is commonly used to detect statistical interactions, however, these traditional parametric statistical methods are less practical for genetic studies attempting to examine possible interactions of multiple genetic variants, for several reasons. First, when high-order interactions involving high-dimensional data are considered, there may be many sparse or empty cells, resulting in inaccurate parameter estimates and an increased type I error. Second, as each additional main effect is included in the model, the total number of parameters (especially for interaction terms) grows exponentially, resulting in too many degrees of freedom and increased type I and type II errors. Third, the lack of a simple pattern of dominant, additive or recessive effect of alleles for common complex traits makes it nearly impossible to model the interaction terms. To address these issues, the non-parametric QMDR approach was proposed. The major advantage of QMDR is that, by effectively reducing the combinations of multi-locus genotypes from high dimensions to one dimension, it avoids the issues of sparse data cells and greatly reduces the degree of freedom necessary for modeling higher-order interactions that can cause problems for traditional parametric statistical methods, thus facilitating the simultaneous detection and characterization of multi-locus interactions and retaining reasonable power even with relatively small sample size. In addition, QMDR does not require any assumption on genetic model, thus it can be more useful to study complex trait, in which the mode of genetic inheritance is usually unknown a prior.

Employing the QMDR approach, the present study identified one significant four-way interaction model (rs174675, rs174697, rs1076560, and rs4436578) associated with verbal fluency, one significant four-way interaction model (rs174675, rs4818, rs1076560, and rs4648317) associated with verbal flexibility, as well as one significant three-way interaction model (rs5993883, rs4648319, and rs4648317) associated with figural flexibility. Because QMDR combines both cross-validation testing and permutation testing to select the best model from all possible two-, three- and four-way models, the problem of multiple testing that inflates the type I error rate in single SNP and haplotype analysis does not apply to the results of QMDR analysis. These three interaction models therefore provide more convincing evidence that *COMT* and *DRD2* are involved in creative potential and act in coordination to contribute to creative potential. In addition, it is worth noting that, among SNPs involved in these three interaction models, only *DRD2* rs1076560 showed a nominal main effect on verbal fluency (Zhang et al., 2014), other SNPs did not demonstrate a main effect on the corresponding DT scores in single SNP analysis. Therefore, this finding further suggests that the underlying genetic mechanism of creativity might be complex and genetic variants without main effect may also contribute to creativity by their interaction effects.

It is intriguing to note that, although both *COMT* and *DRD2* are key genes involved in DA transmission, they are likely to be related to different DA pathways: *COMT* to the prefrontal pathway whereas *DRD2* to the striatal pathway. Accumulating evidence has suggested that these two different pathways may subserve distinct cognitive processes, with DA in the PFC promoting cognitive stability by increasing distractor resistance, while conversely, DA in the striatum promoting cognitive flexibility by allowing the updating of newly relevant representations (Cools and D'Esposito, 2009). Interestingly, although cognitive stability and flexibility are functionally opposed, according to the recent dual-pathway model of creativity, both of them contribute to creativity and creative performance arises from the interaction between them (De Dreu et al., 2008; Nijstad et al., 2010). The model suggests that, while cognitive flexibility facilitates creative potential by establishing more categories of idea and thus contributing to flexibility and fluency, cognitive stability also promotes creative potential by generating many ideas within a few categories and thus contributing to fluency. And most importantly, in the course of ideation people may dynamically employ both the stable processing mode and the flexible processing mode. Based on this notion, it is reasonable to expect that the observed interaction between *COMT* and *DRD2* may implicate individual differences in regulating the dynamic interplay between prefrontal and striatal networks during ideation. Individuals with different combinations of *COMT* and *DRD2* genotypes may vary in the degree to which the stable processing mode and the flexible processing mode are used as well as the patterns how the dynamic balance between these two processing modes is modulated, thus resulting in differences in ideation fluency and flexibility. However, given that the biological functions of the majority of the SNPs included in these interaction models are largely unknown, such explanation remains highly speculative. The specific mechanisms by which these interactions actually work cannot be elucidated in the present study. Further studies integrating both functional analysis and cognitive neuroscience approach will help clarify the detailed biological mechanisms of these interactions.

There are limitations to the present study. First, although the largest such study to date, the moderate sample size of the present study is not as large as would be ideal. Furthermore, since both allele frequencies and LD patterns vary greatly across ethnic populations and only one ethnic group was examined in the present study, the generalization of these findings to other populations is limited. Thus, further prospective studies using larger sample size and replication studies in other ethnic populations are warranted to confirm these suggestive findings. Second, although three significant interaction models between *COMT* and *DRD2* were identified in the present study, sorting out the nature of the interactions in high-dimensional space to infer function remains an interpretive challenge. Functional analysis of these genotypic combinations would be needed to help elucidate these effects. Third, other crucial genes in DA transmission, such as *DRD4* (Mayseless et al., 2013) and *DAT*, were not examined in the present study. Since the regulation of DA transmission is a complex network involving multiple process, full genetic contribution of DA related variants to creativity will rely on far more complex interactions of multiple DA receptors (e.g., *DRD1*, *DRD2*, and *DRD4*), transporters and enzymatic polymorphisms (e.g., *DAT*, *COMT*, *MAOA*, and *MAOB*). Further studies systematically involving such interactions are needed to obtain a clearer overview of DA transmission.

In conclusion, the present study provides preliminary evidence for the involvement of *COMT* as well as the interaction between *COMT* and *DRD2* in creative potential. Although the underlying mechanisms still need to be further investigated, this exploratory study may provide important information to elucidate the contribution of DA related genes to creative potential, which will undoubtedly lead to a better understanding of the underlying genetic basis of creativity.

## Author contributions

Shun Zhang and Jinghuan Zhang were involved in the conception and design of the work. Shun Zhang and Muzi Zhang were involved in data collection. Shun Zhang analyzed the data. Shun Zhang and Jinghuan Zhang contributed in writing the main manuscript text.

### Conflict of interest statement

The authors declare that the research was conducted in the absence of any commercial or financial relationships that could be construed as a potential conflict of interest.
